# Anal canal duplication with heterotopic gastric mucosa and anal stenosis: first case report and literature review

**DOI:** 10.3389/fped.2024.1452116

**Published:** 2024-09-05

**Authors:** Chen Liu, Chuanzhen Xu, Xiaoliang Xu, Yan Zhang, Lei Geng, Yanhui Mei, Hong Ji, Tingliang Fu, Guojian Ding

**Affiliations:** ^1^ Department of Surgery, Shanghai Children’s Hospital, School of Medicine, Shanghai Jiaotong University, Shanghai, China; ^2^ Department of Pediatric Surgery, Binzhou Medical University Hospital, Binzhou, Shandong, China; ^3^ Department of Burn and Reconstructive Surgery, Binzhou Medical University Hospital, Binzhou, Shandong, China; ^4^ Department of Urology, Binzhou Medical University Hospital, Binzhou, Shandong, China; ^5^ Department of Pathology, Qilu Hospital (Qingdao), Cheeloo College of Medicine, Shandong University, Qingdao, Shandong, China

**Keywords:** anal canal duplication, gastrointestinal duplication, heterotopic gastric mucosa, mucosectomy, surgery, children

## Abstract

**Introduction:**

Anal canal duplication (ACD) is a rare entity of gastrointestinal duplication that may be asymptomatic or present complications, such as abscess, fistulae, or malignant changes. The diagnosis and rational management of ACD still need to be clarified.

**Case presentation:**

We present a case of an 18-month-old girl with intractable perianal erosion and painful bowel movements for one year, and chronic constipation for six months. Fistulography revealed a tubular structure (3 cm in length), located posterior to the native anal canal. Mucosectomy was performed through a perineal approach combined with a coccigeal approach, and the postoperative course was uneventful. The pathological findings confirmed the diagnosis of ACD with heterotopic gastric mucosa, a rare combination that has not been described in the literature before. A literature search was conducted on the Medline database for studies reporting ACD in children. The study pool consisted of 77 cases of ACD from 32 studies, including the present case. According to our case report and in line with the literature, 43 cases (55.84%) were incidentally found; the most frequent symptom was constipation (14.29%), followed by painful anal mass or sacral pain (10.39%), and recurrent fistula (7.79%). Coexisting diseases were observed in 31 patients (40.26%), including 19 (24.68%) cases associated with presacral masses. Surgical management was employed in 73 patients (94.81%). ACD excision was performed in 47 patients (64.38%), combined with presacral mass resection or coccygectomy in 19 cases (26.03%).

**Conclusion:**

Preoperative imaging, including fistulography, ultrasonography, and magnetic resonance imaging, can provide useful information, especially for screening its associated anomalies. To prevent potential complications, surgical removal of ACD and associated anomalies is recommended. Mucosectomy may be one of the most effective surgical options for ACD due to its excellent functional outcome.

## Introduction

Intestinal duplication is an uncommon gastrointestinal anomaly. Anal canal duplication (ACD), an extremely rare anomaly, may be asymptomatic or will develop infectious complications, even malignant changes (1–5). ACD may be also associated with congenital anomalies, such as anorectal malformation, anal stenosis, congenital malrotation, presacral teratoma, Currarino triad, urogenital malformation, etc. (6–9). Heterotopic gastric mucosa (HGM) may occur in the digestive tract and other sites, but it rarely occurs in the anorectal region (4, 10–12). Herein, we report an extremely rare case of ACD with HGM and anal stenosis. To the best of our knowledge, this combination has not been reported previously. An English literature review for ACD was conducted, and the diagnosis and management of ACD were discussed to provide useful data with which pediatricians and surgeons diagnose and treat ACD.

## Case presentation

**Figure 1:**
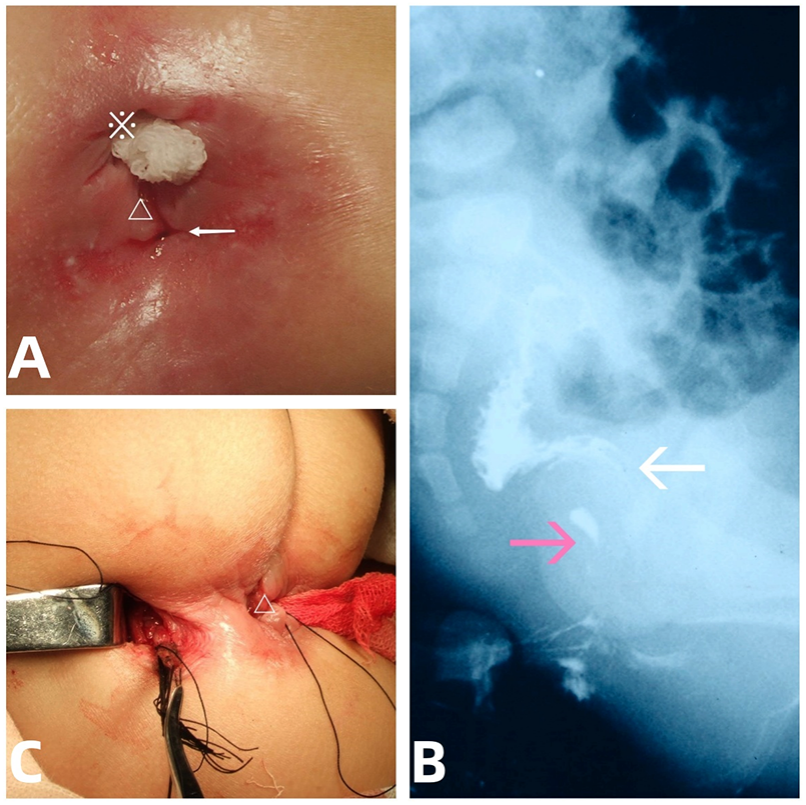
The presentation, fistulogram, and intraoperative view of the ACD. **(A)** Accssory perineal opening with clear fluid discharge (arrow), perianal erosion, anal fissure (△), and native stenosed anus (※). **(B)** Lateral abdominal radiograph showing a contrast medium outlining a tubular duplication (pink arrows) without communication to the native anorectum (white arrows). **(C)** Removal of the tubular mucosa (arrow) via the perineal (△) combined with coccygeal transverse approach.

An 18-month-old girl with a history of painful bowel movements, intractable perineal erosion, and swelling that persisted for one year, and chronic constipation for six months, was admitted to the department of pediatric surgery. Prior to admission, she was diagnosed with perianal eczema and had been treated with a topical ointment, to little effect. Upon physical examination, a perineal orifice of 1.5 mm in diameter (Figure 1A) with clear and colorless fluid discharge, perianal erosion and superficial anal fissures was found on the posterior mid-line of the stenosed native anus.

Contrast injection via both the abnormal orifice and the native stenosed anus revealed a 3- cm-long tubular tract with a blind end, not communicating with the native anorectal lumen (Figure 1B). Abdominopelvic ultrasound (US) findings ruled out presacral mass. An initial diagnosis of ACD associated with anal stenosis was made.

After preoperative anal dilatation and mechanical bowel preparation, the mucosa in the duplicated anal canal was completely excised via a perineal approach combined with a small coccigeal transverse incision (Figure 1C) to avoid damage to the anal sphincter. Perineal anoplasty for the anal stenosis was performed concurrently, and the patient had an uncomplicated postoperative course. The patient was discharged home on postoperative day 6 and had no symptoms related to the previous ACD or anal stenosis at 2**-**year follow**-**up.

**Figure 2:**
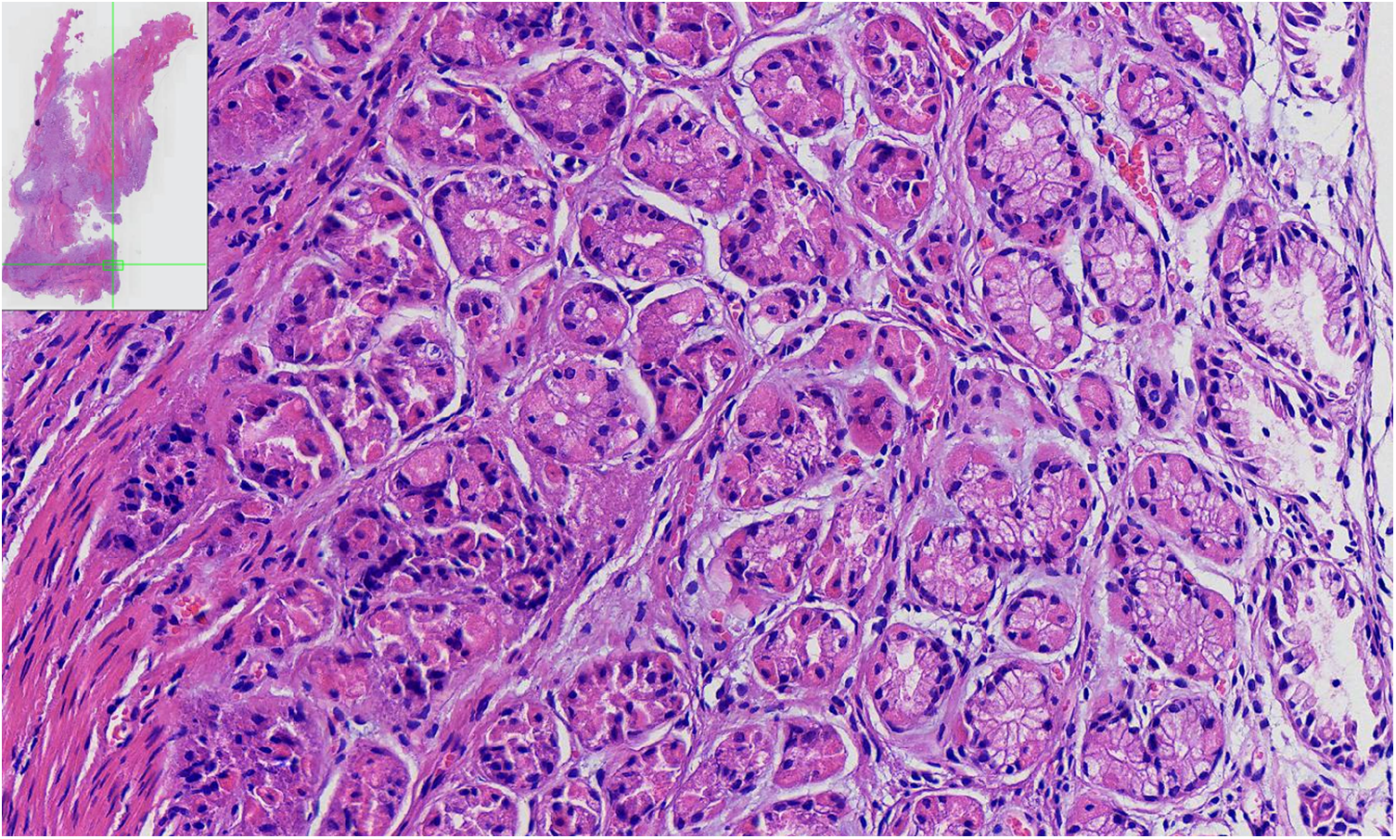
The pathologic findings of the ACD. Pathological findings of the specimen show a tubular structure lined by heterotopic gastric (fundic) mucosa, which consisted of parietal, chief, and mucous neck cells with smooth muscle fibers.

Microscopic examination (Figure 2) revealed a gastric (fundic) mucosa lining in the ACD lumen with smooth muscle cells (SM) in the duplicated anal wall.

A search of the medical literature in English was conducted using the MEDLINE database from 1977 to March 2024. Studies on humans were identified with the terms “anal canal duplication; gastrointestinal duplication; gastric mucosa heterotopia” [as medical subject heading (MeSH) and free**-**text term]; and “rectum” and “anus” (as free**-**text terms). All potentially relevant papers were obtained and evaluated. The variables included age, sex, location, and configuration; symptoms leading to the diagnosis, presence of associated malformations or complications, management (no treatment vs. excision), surgical approach [perineal approaches; perineal and posterior sagittal approaches; sacral and perineal approaches; posterior sagittal anorectoplasty (PSARP)], technique of surgical resection (ACD excision, drainage, and second staged fistulectomy, presacral mass resection, anoplasty, excision of teratoma with coccygectomy); and clinical outcome.

## Results

The study pool consisted of 77 cases of ACD, including two triplications of the anus, from 32 studies (3, 5–9, 13–32) and the present case (Table 1). The median age was 11 months (interquartile range, 3.5 month–3.25 years) and ranged widely from the second day after birth to 18 years of age. Forty-four patients were aged 1 year or younger, while 33 cases were aged beyond 1 year. A total of 68 patients (88.31%) were female.

**Table 1:** Demographic and ACD features according to the symptom categories (*n* = 77).

Variables	Number of cases (*n*)	Percent (%)
Sex		
Male	9	11.69
Female	68	88.31
Age at diagnosis (yrs)		
≤ 1 year	44	57.14
> 1 year	33	42.86
Symptomatolgy		
Asymptomatic and incidental found	43	55.84
Symptomatic	34	44.16
Painful anal mass or sacral pain	8	
Constipation	11	
Recurrent fistula	5	
Iatrogenic fistula	1	
Meningitis	1	
Perianal pruritus	1	
Mucous discharge + constipation	1	
Clear and colorless fluid discharge + constipation	1	
Constipation + presacral abscess	1	
Local infection + septicemia	1	
Abdominal pain + diarrhoea	1	
Abdominal pain	1	
Abdominal pain, anal mucous secretion	1	
External orifice and location		
Not visible	2	2.60
Visible (lithotomy position)	75	97.40
6 o’clock	75^a^	97.40
7 o’clock	2	2.60
Configuration		
Tubular (length, mm)	67^a^ (median, 10 mm)	87.01
5–9 mm	3	4.48
10–19 mm	34	50.75
20–29 mm	14	20.90
30–35 mm	12	17.91
Not available	4	5.97
Cystic (size, mm)	12	15.58
Communication with the anorectal lumen		
Yes	3	3.90
No	74	96.10
Associated malformations	31	40.26
Presacral mass	19	24.68
Mature teratoma	9	
Presacral cyst	6	
Cystic hemartoma	1	
Ependymoma	1	
Demoid cyst with infection	1	
Intrasacral meningocele	1	
Ventricular septal defect	1	
Malrotation	2	
Anal stenosis	2	
Duplex kidney	2	
IND type B	1	
Currarino triad + pelvocaliectasis	1	
Spina bifida aperta	1	
Cleft and lip palate,giant omphalocele, complex genitourinary malformations, hypoplastic kidney	1	
Ureteric duplication	1	
Method of diagnosis		
US	36	46.75
MRI	32 (positive finding 17)	41.56
Fistulography	25	32.47
CT scan	2	2.60
Surgical probe	2	2.60
Inspection	2	2.60
Therapeutic options		
Surgical procedure	75^a^	94.81
ACD excision	49^a^	
ACD excision + presacral mass resection	17	
ACD + removal of teratoma and coccygectomy	2	
ACD excision with anoplasty	2	
ACD excision/drainage and staged fistulectomy	1	
No treatment	4	5.19
Surgical approach		
Perineal	57^a^	
Perineal + posterior saggital	15	
Perineal + coccygeal	1	
Not available	2	
Histopathology		
Squamous epithelium in the distal end	51	
Translational epithelium lining in the proximal end	30	
Smooth-muscle cells in the duplicated anal canal wall	29	
Pseudostratified columnar epithelial lining	9	
HGM with smooth muscle cells	The present case	
Not available	12	

ACD, anal canal duplication; IND, intestinal neuronal dysplasia; US, ultrasound; MRI, magnetic resonance imaging; CT, computerized tomography; HGM, heterotopic gastric mucosa.

^a^ Including 2 cases with anal canal triplication.

Among all the cases, 43 cases (55.84%) were incidentally found; The clinical presentation included constipation in 11 cases (14.29%), painful anal mass or sacral pain in 8 cases (10.39%), and recurrent or iatrogenic fistula in 6 cases (7.79%). Others included meningitis, perianal pruitus, mucous discharge + constipation, constipation + presacral abscess, local infection + septicemia, abdominal pain + diarrhoea, abdominal pain + anal mucous secretion. Our case presented clear and colorless fluid discharge, erosion, and constipation.

With the patient in lithotomy position, the orifice was posteriorly located in the midline (at “6 o’clock”, 75 orifices) or more laterally located (“7 o’clock”, 2 orifices), except in two cases with an invisible external orifice. Investigation methods included abdominopelvic US in 36 cases (46.75%); MRI in 32 cases (41.56%, positive finding in 17 cases); fistulography in 25 cases (32.47%); and computerized tomography (CT) in 2 cases (2.60%).

The ACD configuration was redefined as tubular in 67 cases (87.01%), including one case with a distal partial cyst or cystic structure in 12 cases (15.58%). The median length of tubular ACD was 10 mm (in 69 orifices of 67 cases) and ranged between 5 mm and 35 mm, including 5–9 mm (3 cases), 10–19 mm (34 cases), 20–29 mm (14 cases), and 30–35 mm (12 cases). The cysts ranged from 1.0 to 5.0 cm in diameter, including 6 cysts of 5 cm in diameter.

Coexisting diseases were observed in 31 patients (40.26%). Nineteen cases (24.68%) were associated with presacral mass, including mature teratoma (9 cases), presacral cyst (4 cases), cystic hemartoma (one case), minor intrasacral meningocele (one case), and demoid cyst with infection (one case). Others included anal stenosis, congenital malrotation, duplex kidney, anorectal malformation, spina bifida aperta L_5_ with sacro-coccygeal teratoma, ventricular septal defect, Currarino triad (a tethered spinal cord, hemisacrum, presacral mature teratoma) and left pelvocaliectasis, ureteric duplication, IND type B with presacral ependymoma, cleft and lip palate, giant omphalocece, and complex genitourinary malformations, hypoplastic kidney, lumbosacral myelomeningocele (previously treated at birth) with presacral mature teratoma.

Surgical management was employed in 73 patients (94.81%). ACD excision was performed in 47 patients (64.38%), combined with presacral mass resection or coccygectomy in 19 cases (26.03%). Surgical approaches included simple perineal in 55 patients (75.34%), and a combination of perineal and posterior saggital in 15 patients (20.55%).

The histopathologic findings of the ACD have been reported in 65 cases (84.42%). There are 51 cases with squamous epithelium in the distal end, 30 cases with translational epithelium lining in the proximal end, 29 cases with smooth–muscle cells in the duplicated anal wall, and 9 cases with pseudostratified columnar epithelial lining.

## Discussion

Duplication of the intestinal tract can occur in any part of the digestive tract and is a diverse and complicated spectrum of congenital malformations (2, 33). ACD is the most distal and the least frequent digestive duplication, presenting as a perineal opening in the midline, posterior to the normal anus (3, 6). About 76 cases of childhood have been reported in the English literature (5, 27, 32).

ACD may be due to an abnormal cloaca combined with varied abnormalities, such as presacral mature teratoma or cyst, Currarino triad (a tethered spinal cord, hemisacrum, presacral mature teratoma), meningocele, renal hypoplasia, and anorectal malformations (9, 14, 19, 34–36). It was previously hypothesized that an inferiorly extending anal sinus may lead to an additional lumen of ACD (37). Histopathological features are (1) squamous cells at the caudal end; (2) transitional and columnar epithelium at the cranial portion; and (3) smooth-muscle cells in the wall (6, 12).

ACD may stay asymptomatic, which is incidentally found by the family pediatrician or by a parent (21). However, 44.16% of cases presented symptoms of local or systemic infection, such as epidural abscess with sepsis (5, 15); nonspecific anal symptoms, e.g., constipation, painful anal mass or sacral pain, recurrent perianal fistula, pruritus ani, and mucous discharge from the duplicated anus (15, 29, 30, 35). Our case presented with clear fluid discharge from the abnormal orifice and intractable perianal erosion, which was suggestive of chemical dermatitis. The acid-secreting gastric (fundic) mucosa lining on the inner surface of the ACD was confirmed by histopathological findings. Other clinical presentations in patients with GMH in anus, rectum, or rectal duplication cysts included perianal fistulae, anal peptic ulceration on the opposite side, anorectal bleeding, pyloric gland adenoma, polypoid, Helicobacter pylori colonization, and even adenocarcinoma (10, 11, 38–42). ACD should be considered a differential diagnosis in patients with abscesses, recurrent fistulous tracts, or any other anorectal disorders (27, 43–45). What's more, a combination of ACD and HGM should be considered when making a diagnosis of ACD, although HGM in anorectal duplications is a finding of extreme rarity which gives rise to difficulty in diagnosis and pathogenesis.

Regarding the diagnosis, a perineal orifice in the midline located behind the anus should raise suspicion of ACD (19, 22). In this case series, symptom duration varied from days to months to years before making an accurate diagnosis and our case was initially undiagnosed. It is necessary to expand the information for this rare pathological entity. The importance of a complete pediatric physical examination, including exploration via surgical probe or metal catheter, should be emphasized (17, 27). Imaging examinations included pelvic X–rays, fistulography, barium contrast studies, abdominopelvic US, and MRI (6, 22, 31). Fistulography can reveal a tubular structure or a cystic structure behind the native anal canal. Abdominopelvic US and MRI are considered useful tools to rule out the associated presacral mass (2, 8, 9, 14, 19, 32). An US examination is more suitable to detect presacral mass in infants (46). 99mTc–Pertechnetate scanning can be used in patients with ACD with suspicion of HGM (47, 48).

Regarding the treatment, a few cases with asymptomatic ACD received conservative treatment (17, 19, 35). However, surgical removal of the ACD is essential, even for asymptomatic patients, to prevent inflammatory complications or malignant changes (6, 16, 22, 43). The surgical option (mucosectomy or perineal/posterior sagittal approach) depends on the patient's age, length of ACD, and associated anomalies (2, 33). Anoplasty was performed by suturing the full–thickness native anus to the posterior aspect of the sphincter complex for ACD with anal stenosis. Patients with ACD associated presacral mass need a removal of ACD combined with an excision of presacral mass via a posterior sagittal approach (9). This procedure has been considered a complex surgical challenge that requires a customized plan for rational management (19, 32).

The important step of the procedure is to separate ACD from the posterior rectal wall (19). Complete excision through a perineal or posterior sagittal approach is recommended (14, 19, 22, 28, 29, 32, 34). Tiryaki et al. (28) reported mucosal stripping with primary repair, which is considered a simple and safe technique. It may avoid unnecessary dissection of the sphincter and posterior rectal wall, take less time, and achieve good functional results (26, 28, 29, 34). Our case undergoing mucosal stripping with primary anoplasty via the perineal combined with coccygeal transverse approach had a good postoperative outcome with normal anal sphincter control, as Koga et al. (22) described.

Postoperative complications included a temporary external sphincter insufficiency with fecal incontinence, which was surgically treated with sphincter repair, and an abdominal wound infection in the colostomy site in cases treated earlier (22).

The limitation of this case reports and systemic review is that the included literature over a long period (1970–2022) and the included patients were extracted from small case series or isolated case report. The overall small patient sample made the comparisons between treatment approaches impossible. Larger clinical studies are needed for rational treatment of ACD.

In conclusion, ACD is a rare gastrointestinal anomaly which may remain asymptomatic before surgery or present as complications. Our case, presenting with perianal erosion caused by discharge of gastric acid secretion, may complicate the diagnosis of ACD accompanied by HGM. According to our case report and in line with the literature, the combination of fistulography and abdominopelvic US/MRI can provide useful information for the diagnosis and preoperative assessment of ACD with associated anomalies. To prevent potential complications, surgical removal of ACD with associated abnormalities is recommended. Mucosectomy may be one of the most effective surgical options for ACD due to its excellent functional outcome.

## Data Availability

The raw data supporting the conclusions of this article will be made available by the authors, without undue reservation.
